# An Unusual Case of Obscure Gastrointestinal Bleeding in a Patient with Coeliac Disease

**DOI:** 10.1155/2011/634684

**Published:** 2011-11-13

**Authors:** M. Gwiggner, P. Patel

**Affiliations:** Gastroenterology Service, Southampton General Hospital, Southampton University Hospitals NHS Trust, F Level, Centre Block, Tremona Road, Southampton SO16 6YD, UK

## Abstract

This paper describes the journey of a patient with coeliac disease who presented with overt obscure gastrointestinal bleeding. Upper and lower gastrointestinal endoscopy did not reveal a source of bleeding, but an abdominal CT scan detected abnormal lymphadenopathy and a wireless capsule endoscopy diagnosed a jejunal tumour, which was surgically removed. Gastrointestinal bleeding is rare in celiac disease. Malignant tumours of the small intestine are generally uncommon, but celiac disease represents a significant risk factor. Wireless capsule endoscopy has been a useful tool to investigate patients with obscure gastrointestinal bleeding.

## 1. Introduction

Coeliac disease is a common autoimmune-mediated gluten-sensitive enteropathy which can lead to partial or total villous atrophy of the small bowel. Coeliac disease affects about 0.5–1% of the western world in genetically susceptible people [1, 2]. Treatment consists of strictly following a gluten-free diet. Gastrointestinal bleeding is uncommon in coeliac disease, especially overt gastrointestinal bleeding [3]. Cancers of the small intestine are generally infrequent, but celiac disease represents one of the risk factors to develop malignant disease in the small intestine [2]. Wireless capsule endoscopy has been a useful tool to investigate patients with obscure gastrointestinal bleeding and tumours of the small intestine.

## 2. Clinical Presentation

**Figure 1:**
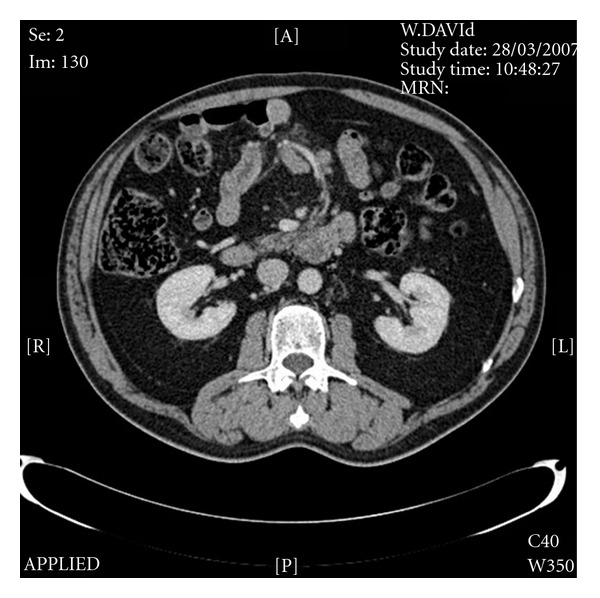
Axial reconstruction of abdominal computed tomography scan shows lymphadenopathy in the region of the superior mesenteric vein.

A 72-year-old patient with a history of coeliac disease, ischaemic heart disease, and atrial fibrillation, for which he was anticoagulated with warfarin, was admitted to hospital with dark red bleeding per rectum. Blood tests revealed a significant normocytic anaemia and a rise in blood urea. He had blood transfused and underwent upper gastrointestinal endoscopy to the second part of the duodenum, which was normal. Warfarin was discontinued, and he attended for an outpatient colonoscopy, which did not reveal a source of bleeding. At this time his haemoglobin was stable and there were no further signs of bleeding and no other gastrointestinal tract symptoms. A computed tomography scan of his abdomen with oral contrast was arranged to look for any obvious small bowel luminal abnormality (Figure 1).

**Figure 2:**
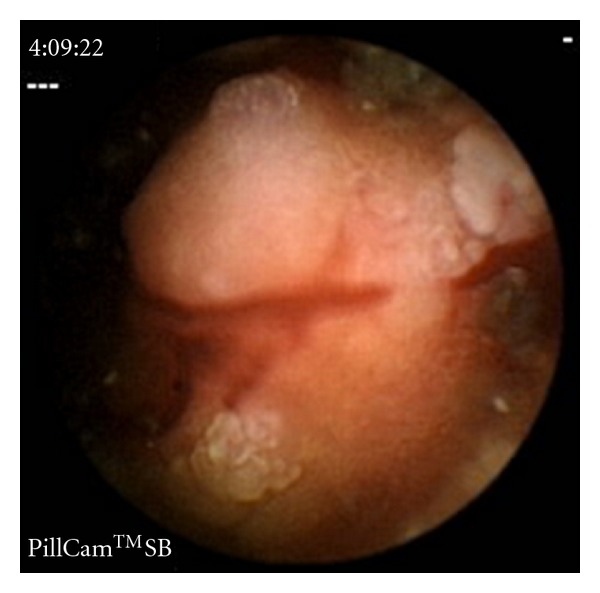
Images from the wireless capsule endoscopy demonstrate a malignant looking jejunal stricture due to a tumour.

The abdominal CT scan showed no luminal abnormality but revealed lymphadenopathy (largest node 13 mm) in the region of the superior mesenteric vein suggesting midgut pathology. No other pathological abnormality was diagnosed. He was referred for a wireless capsule endoscopy, which discovered a jejunal tumour causing a malignant looking stricture preventing the impacted wireless capsule to pass and resulted in small bowel obstruction requiring a laparotomy (Figure 2).

**Figure 3:**
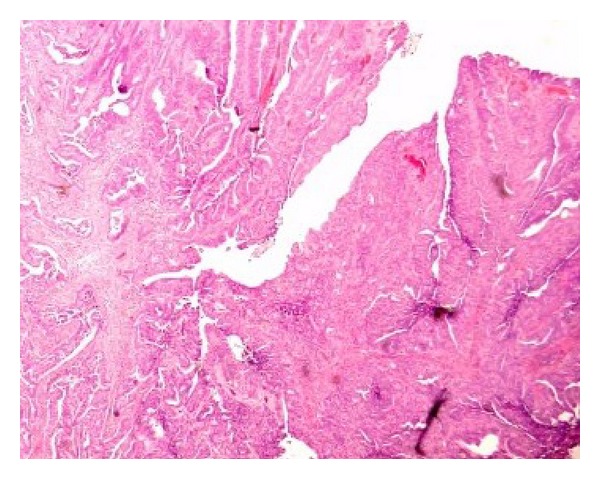
Histology of the resected specimen shows moderately differentiated adenocarcinoma.

 The laparotomy confirmed the jejunal tumour, and the patient underwent a resection with end-to-end anastomosis of the jejunum. Lymph nodes were not removed at the time of surgery as the tumour was assumed to be a lymphoma in view of his past history of coeliac disease. Histopathological examination of the resection specimen demonstrated a moderately differentiated adenocarcinoma with clear resection margins, rather than a lymphoma (Figure 3).

 MRI of the liver showed a single metastasis in segment 6. The patient was undergoing palliative chemotherapy but sadly passed away.

## 3. Discussion

Coeliac disease affects about 0.5–1% of the western world in genetically susceptible people [1, 2] and can lead to iron-deficiency anaemia due to malabsorption, but gastrointestinal bleeding, especially overt gastrointestinal bleeding, is rare. Mant et al. conducted a study measuring the stool blood loss directly by using 51Cr radiolabeled red cells in 18 consecutive untreated patients with coeliac disease and found that only 1 in 18 patients had blood loss of more than 1.5 mL per day indicating a very low prevalence of significant gastrointestinal bleeding in coeliac disease [3]. 

Wireless capsule endoscopy has become a useful tool to investigate the small intestine. The commonest lesions diagnosed by capsule endoscopy in 385 patients were small bowel ulcers and erosions, followed by tumours and arteriovenous malformations, with a diagnostic yield of 58% for definite lesions [4]. Pennazio et al. studied 100 consecutive patients undergoing capsule endoscopy of which 26 patients had on-going overt bleeding, 31 with previous overt bleeding, and 43 with guaiac-positive stools and iron- deficiency anaemia. The yield of positive findings on capsule endoscopy was 92.3%, 12.9%, and 44.2%, respectively [5].

Tumours of the small bowel represent only 1–5% of all gastrointestinal malignancies, which is striking as the small intestine accounts for 75% of the length and 90% of the mucosal surface area of the gastrointestinal tract [6]. The most common presentation of small bowel tumours is occult gastrointestinal bleeding, followed by abnormal imaging studies [7]. Occult gastrointestinal bleeding, especially overt occult gastrointestinal bleeding in coeliac disease, should therefore be investigated promptly. There are several precursor states for small intestinal adenocarcinoma which include familial polyposis syndromes, coeliac disease, and Crohn's disease [6]. Howdle et al. collected clinicopathological data for 395 cases of small bowel tumours, including 175 adenocarcinomas, 107 lymphomas, and 79 carcinoid tumours. In 13% of adenocarcinoma cases and in 39% of lymphomas, there was a previous diagnosis of coeliac disease. Survival rates at 30 months for adenocarcinoma, lymphoma, and carcinoid tumours were 58%, 45%, and 78%, respectively [8].

In addition to small bowel lymphomas, coeliac disease is also a recognised risk factor for small bowel adenocarcinomas. Small bowel tumours should therefore be considered early in coeliac patients with obscure gastrointestinal bleeding as lymphomas as well as adenocarcinomas carry a poor prognosis. Wireless capsule endoscopy has a high diagnostic yield in overt obscure gastrointestinal bleeding, especially in the acute phase, and should be considered without delay. 

## References

[1] Arber N, Neugut AI, Bernard Weinstein I, Holt P (1997). Molecular genetics of small bowel cancer. *Cancer Epidemiology Biomarkers and Prevention*.

[2] Neugut AI, Jacobson JS, Suh S, Mukherjee R, Arber N (1998). The epidemiology of cancer of the small bowel. *Cancer Epidemiology Biomarkers and Prevention*.

[3] Mant MJ, Bain VG, Maguire CG, Murland K, Yacyshyn BR (2006). Prevalence of occult gastrointestinal bleeding in celiac disease. *Clinical Gastroenterology and Hepatology*.

[4] Goenka MK, Majumder S, Kumar S, Sethy PK, Goenka U (2011). Single center experience of capsule endoscopy in patients with obscure gastrointestinal bleeding. *World Journal of Gastroenterology*.

[5] Pennazio M, Santucci R, Rondonotti E (2004). Outcome of patients with obscure gastrointestinal bleeding after capsule endoscopy: report of 100 consecutive cases. *Gastroenterology*.

[6] Green PH, Jabri B (2002). Celiac disease and other precursors to small-bowel malignancy. *Gastroenterology Clinics of North America*.

[7] Lee BI, Choi H, Choi K-Y (2011). Clinical characteristics of small bowel tumors diagnosed by double-balloon endoscopy: KASID multi-center study. *Digestive Diseases and Sciences*.

[8] Howdle PD, Jalal PK, Holmes GK, Houlston RS (2003). Primary small-bowel malignancy in the UK and its association with coeliac disease. *QJM*.

